# Generation of polycyclic aromatic hydrocarbons (PAHs) during woodworking operations

**DOI:** 10.3389/fonc.2012.00148

**Published:** 2012-10-18

**Authors:** Evin D. Bruschweiler, Brigitta Danuser, Cong Khanh Huynh, Pascal Wild, Patrick Schupfer, David Vernez, Philippe Boiteux, Nancy B. Hopf

**Affiliations:** ^1^Institute for Work and Health (IST), University of LausanneLausanne, Switzerland; ^2^Institute for Research and SafetyVandoeuvre les Nancy, France

**Keywords:** wood dust, polycyclic aromatic hydrocarbons, occupational exposure, sinonasal cancer, wood operations

## Abstract

Occupational exposures to wood dust have been associated with an elevated risk of sinonasal cancer (SNC). Wood dust is recognized as a human carcinogen but the specific cancer causative agent remains unknown. One possible explanation is a co-exposure to; wood dust and polycyclic aromatic hydrocarbons (PAHs). PAHs could be generated during incomplete combustion of wood due to heat created by use of power tools. To determine if PAHs are generated from wood during common wood working operations, PAH concentrations in wood dust samples collected in an experimental chamber operated under controlled conditions were analyzed. In addition, personal air samples from workers exposed to wood dust (*n* = 30) were collected. Wood dust was generated using three different power tools: vibrating sander, belt sander, and saw; and six wood materials: fir, Medium Density Fiberboard (MDF), beech, mahogany, oak and wood melamine. Monitoring of wood workers was carried out by means of personal sampler device during wood working operations. We measured 21 PAH concentrations in wood dust samples by capillary gas chromatography-ion trap mass spectrometry (GC-MS). Total PAH concentrations in wood dust varied greatly (0.24–7.95 ppm) with the lowest being in MDF dust and the highest in wood melamine dust. Personal PAH exposures were between 37.5–119.8 ng m^−3^ during wood working operations. Our results suggest that PAH exposures are present during woodworking operations and hence could play a role in the mechanism of cancer induction related to wood dust exposure.

## Introduction

Breathing wood dust from processes such as sanding, sawing, and cutting is common in occupational settings affecting an estimated 3.6 million workers across Europe (Kauppinen et al., 2006). Included in this survey were typical woodworking occupations such as construction and bench carpenter, woodworking machine operator, sawer, cabinet maker, and joiner. Exposures to wood dust may cause respiratory health problems such as allergic upper airway disease (asthma), non-allergic pulmonary disease, and lung disorders (Jacobsen et al., 2010). Although the more severe health effect is cancer; adenocarcinomas of nasal cavity and paranasal sinuses sinonasal cancer (SNC). The International Agency for Research on Cancer (IARC) has classified wood dust as carcinogenic to humans (Group 1), based on the association of wood dust exposure with elevated SNC risk. Wood dust exposures were principally to hardwood dusts such as beech or oak (IARC, 1995). Although wood dust is recognized as a human carcinogen, its carcinogenic mechanisms and the specific cancer causative agent i.e., wood dust component, wood dust with chemical additives and/or physical properties of wood dust, remain unknown (Nylander and Dement, 1993).

Wood is a complex substance composed mainly of cellulose, hemicelluloses, lignin, and a large number of substances of a lower relative molecular mass. These include organic compounds (terpenes, resin acids, fatty acids, alcohols, sterol, steryl esters, and glycerides), polar organic compounds (tannins, flavonoids, quionones, and lignans), and water soluble compounds [carbohydrates, alkaloids, proteins (IARC, 1995)]. Wood workers are exposed not only to wood dust but also to fungus, and chemicals such as formaldehyde, wood preservatives, and glues. Because exposures are to mixtures, it is difficult to determine the specific cancer causative agents. Additionally, IARC suggested that workers are not only exposed to wood dust but to pyrolysis products at the same time. During sawing operations, pyrolysis of wood may occur due to the increase of heat from friction, and generate polycyclic aromatic hydrocarbons (PAHs) such as benzo[a]pyrene (BaP) (IARC, 1981). PAHs are formed during incomplete combustion of organic matter such as petroleum, oil, coal, and wood (Hertel, 1998). Several PAHs are carcinogenic: BaP is carcinogenic to humans (Group 1); benz[a]anthracene, dibenz[a,h]anthracene are probable human carcinogens (Group 2A), and benzo[b]fluoranthene, benzo[j]fluoranthene, benzo[k]fluoranthene, dibenzo[a,e]pyrene, dibenzo[a,h]pyrene, dibenzo[a,i]pyrene, dibenzo[a,l]pyrene, and indeno[1,2,3-cd]pyrene are possible human carcinogens (Group 2B) (IARC, 2010).

PAHs are often adsorbed to particles (Boffetta et al., 1997), making large wood dust particles a vehicle for PAHs. Associations between occupational PAH exposures and SNC have been observed in different industry sectors such as non-ferrous metal basic industries, industrial chemicals manufacturing, iron and steel basic industry, printing, publishing and allied industry, manufacturing of wood, and cork products (Rushton et al., 2010). To assess cancer risks in the wood processing sector, Kauppinen et al. studied occupational exposures to chemical agents among plywood woodworkers (Kauppinen, 1986). Their hypothesis was that formation of PAHs occurs by friction heat during sawing and sanding of plywood. Only two dust samples were collected for PAH analysis, one for each operation. They detected traces of PAHs; however, they concluded that the likely PAH source was the forklift exhaust gases but did not rule out the possibility of other sources, specifically from the wood processing itself (Kauppinen, 1986).

Occupational exposures limits (OELs) for wood dust exist in many countries; however, the limits differ greatly. The American Conference of Governmental Industrial Hygienists (ACGIH) established a threshold limit value (TLV) of 1 mg m^−3^ for all wood types except red cedar (TLV 0.5 mg m^−3^) (ACGIH, 2012). The Swiss OELs were 2 mg m^−3^ for hardwood and 5 mg m^−3^ for softwood inhalable dust until 2009, when an OEL of 2 mg m^−3^ was established for both types of wood dust (SUVA, 2009). Some countries differentiate between hardwood and softwood in their regulations; however, hardwood is not necessarily denser than softwood, as often construed. This classification is botanically based on the cells structure in the wood species, and do not refer to the wood's hardness. Wood dust particles produced during wood working operations usually have aerodynamic diameters greater that 10 μm (Whitehead et al., 1981; Pisaniello et al., 1991; Harper et al., 2004; Lee et al., 2011). A high proportion of these large particles are deposited in the nasal cavity, and may be involved in the development of SNC (NTP, 2000).

Our aim in this study was to measure PAH concentrations in dust generated from wood materials (fir, mahogany, beech, oak, Medium Density Fiberboard (MDF), wood melamine) during common wood working operations (vibration sander, belt sander, and saw) in an experimental chamber (a), and among wood workers in different wood working processing factories (b).

## Materials and methods

### Experimental study

#### Experimental chamber

To simulate wood working operations under controlled conditions, the experiments were carried out in an experimental chamber (10 m^3^) as previously described (Guillemin, 1975). This chamber's air exchange rate was fixed at 10 times per hour, which is similar to a well ventilated room. The experimental chamber was kept under slight positive pressure (5 Pa) to avoid contamination by air outside the chamber. To avoid cross contamination between different experiments, the chamber was vacuum cleaned to remove all wood dust and ventilated for 2 h prior to the next experiment.

#### Wood materials

We selected four commonly used wood types; fir, beech, sipo mahogany (sipo), and oak; and two commonly used wood materials: MDF and wood melamine (wooden boards). Fir represents softwood, beech, and oak hardwood. Sipo is an exotic wood used in the furniture industry. Wooden boards are manufactured by mixing wood fibers with a resin, and milled to form sheets. Wood melamine differs from MDF by having papers impregnated with thermosetting plastic resin lining both sides of the board.

#### Wood operations

We generated wood dust using three different power tools: a handheld vibration sander fitted with 120 grade sandpaper (11,000 revolutions min^−1^, 280 W); a handheld belt sander fitted with 120 grade sandpaper (480 revolutions min^−1^, 1200 W), and a circular saw (4200 revolutions min^−1^).

To quantify PAH concentrations in different materials, we generated wood dust from all six wood materials by vibration sanding. To quantify PAH concentrations in dust generated during different wood working operations, we included two additional operations: sawing and belt sanding using only three wood materials: fir, oak, and wood melamine. For all experiments, the wood material (40 × 20 cm) was clamped to a table (85 × 75 cm) placed inside the experimental chamber. Dust was generated during 3 h (including breaks) by the same operator. The power tool was operated during 30 min intervals and repeated four times, except for the sawing operation. Sawing was performed during 15 min four times only because insufficient wood material remained to continue sawing. Grab dust samples were collected from the table top after suspended particles had settled (30 min) by scooping up the dust with one hand into a collection plastic bag. These grab samples are settled dust samples and differ in particle size distribution compared to dust collected in air.

### Field study

#### Study population

Thirty construction (parquet layers, installers, and carpenters) and furniture workers located in Bern, Fribourg, Lausanne, and Geneva (Switzerland) were recruited. This study was conducted between December 2010 and January 2012. Workers were classified into two exposure groups: “traditional factory workers” (*n* = 16) using common wood working tools; and “modern furniture industry workers” (*n* = 14) using an automated (Computer Numerically Controlled) wood router, which was computerized for sawing, sanding, cutting, making joints, etc. In the modern factory, machines and manual work stations were all equipped with local exhaust ventilation (LEV). In addition, the settled dust fell down into grid protected wells in the floor, which reduced the chance of re-suspending settled dust. This study is approved by the Ethics Committee of the Faculty of Biology and Medicine at the University of Lausanne. We obtained an informed consent from each participant before sampling.

#### Personal inhalable dust monitoring

Personal air sampling was used to collect dust during two consecutive work shifts for all workers. A 37 mm closed-face cassette (CFC) sampler equipped with glass fiber filters (GF/B, Ø37 mm, Whatman) was used, and operated with a flow rate of 2 L min^−1^ (Esscort ELF pump, MSA, Pensylvania, USA). The CFC samplers were placed in the workers' breathing zone during collection.

### Gravimetric analysis

Wood dust concentrations were determined by gravimetric analysis. The GF/B filters were conditioned 24 h at constant relative humidity (%RH) prior to weighing on a microbalance with a readability of ±1 μg (Satorius, Model M5P, IG Instrumenten Gesellschaft AG, Switzerland) before and after sample collection using a glove box. Humidity was regulated by a saturated salt bath of Ca (NO_3_)_2_·4H_2_O (55% RH at 20°C).

### Particles size analysis

Particle size distributions of wood dust were determined for six workplaces (four traditional and two modern). A multi-stage cascade impactor (model 1, ACFM, nine stages, Anderson Inc., USA) equipped with eight glass fiber filter stages and a backup filter (Glass fiber filter 934AH, pore size 1.5 μm, Ø81 mm, Whatman) was used to collect particles with aerodynamic diameters from <0.4 to >11 μm. The collection flow rate was 28.3 l min^−1^ [1 cubic foot per minute (CFM)]. The mass concentrations for each stage were determined by gravimetric analysis. Mass median aerodynamic diameter (MMAD) and geometric standard deviation (GSD) of the wood dust were determined using the method developed by O'Shaughnessy and Raabe (2003).

### PAH analysis

PAH concentrations in dust samples were determined as previously described by Vu Duc and colleagues (Vu-Duc et al., 1995, 2007). In brief, wood dust samples were soxhlet extracted (24-h) with toluene, then liquid-liquid extracted (cyclohexane and dimethylformamid), followed by micro-column purification. Samples were analyzed with a capillary gas chromatography-ion trap mass spectrometry (GC-MS). The GC (Varian Saturn 2000 MS) was equipped with a fused silica column (50% polyphenylsilicone phase, 30 m × 0.25 mm, SGE, Infochroma, Switzerland) and helium as the carrier gas. The MS detection was operated in the electron ionization (EI) mode (50 eV) (2 scans/s; 55–350 m/z). A mixture of 36 PAHs in toluene (SRM 2260a National Institute of Standards and Technology, NIST, USA) was used as the calibration standard. Indeno[1,2,3-cd]fluoranthene was used as an internal standard (IS). Identification of PAHs was performed by comparing the peaks to known standards, and quantification by using the peak ratio relative to the IS. The limit of detection (LOD) was determined for each dust sample, and was typically 0.001 ppm. The limit of quantification (LOQ) was 0.032 ng per injection (1 ul). Recovery was 90.7–101.4%.

Table 1 lists all PAHs quantified and their carcinogenic (IARC) classifications (IARC, 2010); Group 1 “carcinogenic to humans,” Group 2A “probably carcinogenic to humans,” and Group 2B “possibly carcinogenic to humans,” Of the 21 PAHs identified, we have summed 6 PAHs (Σ6PAHs) from group 1, 2A, and 2B, and present them here as “potentially carcinogenic PAHs.” The Σ6PAHs included the following PAHs: benzo[a]pyrene, naphthalene, benz[a]anthracene, benzo[b]fluoranthene, benzo[k]fluoranthene, indeno[1,2,3-cd]pyrene.

**Table 1:** **PAHs analyzed, number of aromatic rings in the chemical structure (No. aromatic rings), molecular weight (MW), and their IARC classifications; Group 1 “carcinogenic to humans,” Group 2A “probably carcinogenic to humans,” and Group 2B “possibly carcinogenic to humans”**.

**PAH**	**MW**	**No. aromatic rings**	**IARC Group**
Naphthalene	128	2/3	2B
Fluorene	166	2/3	3
Phenanthrene	178	2/3	3
Anthracene	178	2/3	3
Fluoranthene	202	4	3
Pyrene	202	4	3
Benzo[a]fluorene	216	4	3
Benz[a]anthracene	228	4	2A
Chrysene	228	4	3
Benzo[b]fluoranthene	252	5	2B
Benzo[k]fluoranthene	252	5	2B
Benzo[j]fluoranthene	252	5	2B
Benzo[e]pyrene	252	5	3
Benzo[a]pyrene	252	5	1
Perylene	252	5	3
Benzo[ghi]perylene	276	6	3
Indeno[1,2,3-cd]pyrene	276	6	2B
Benzo[b]chrysene	278	6	3
Dibenzo[a,j]anthracene	278	6	
Dibenzo[a,h]anthracene	278	6	2A
Dibenzo[a,c]anthracene	278	6	

### Statistical analysis

One or two factor analyses of variance of the log-transformed concentrations were used when comparing groups. For each significant factor from ANOVA the groups were compared *post-hoc* using Holm's multiplicity adjustment. All statistical analyses were performed using STATA software (StataCorp. 2011. Stata Statistical Software: Release 12. College Station, TX: StataCorp LP).

## Results

### Experimental study

#### Wood materials

**Figure 1:**
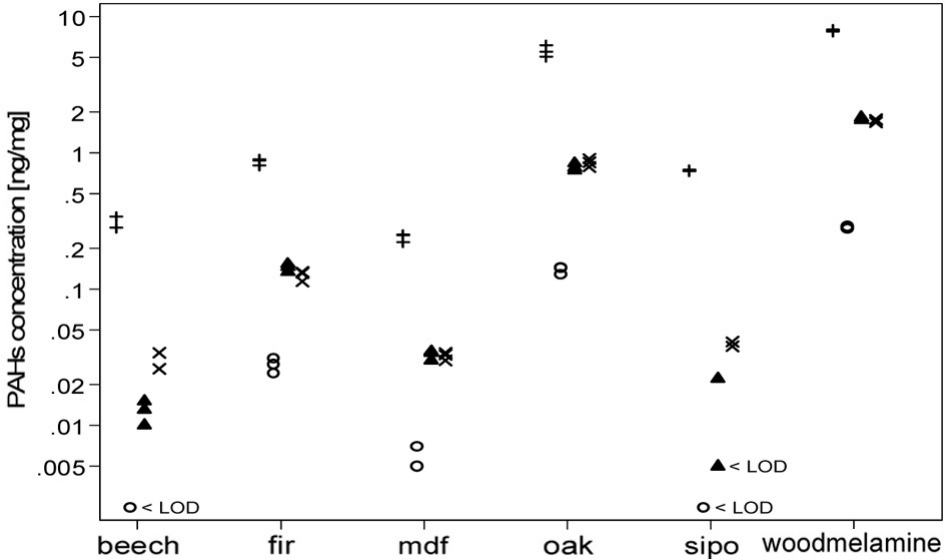
**Concentrations of total PAHs (crosses +), BaP (open circles °), Σ6PAHs (filled triangles ▲), and pyrene (x) according to wood material generated during vibration sanding in the exposure chamber.** < LOD = below the limit of detection.

We observed a difference in total PAH concentrations between the wood materials studied in the experimental chamber (Figure 1). Three samples were obtained for each wood material. The lowest total PAH concentration was measured in MDF dust and contained mainly non-carcinogenic PAHs (fluorene, phenanthrene, fluoranthene, and pyrene). Total PAH concentrations found in dust from beech and sipo, were not significantly different from MDF. The total PAH concentration in fir dust was higher than MDF and contained traces of BaP. Oak dust contained the second highest total PAH concentration, and had quantifiable levels of some carcinogenic PAHs: BaP, benz[a]anthracene, benzo[b]fluoranthene, and benzo[j]fluoranthene. Wood melamine dust contained the highest concentration of total PAHs compared with all other wood materials (MDF, beech, fir, sipo, and oak). Wood melamine differs from the other wood materials as it is a heterogeneous material containing plastic resin and glue. We detected the presence of 21 PAHs, which combined gave the highest concentration of total PAHs for wood melamine. The BaP percentage ([BaP]/[total PAHs] × 100) varied across wood materials with 2.3–3.5% for fir, MDF, oak, and wood melamine; and >1% for sipo and beech. Concentrations for total PAHs, Σ6PAHs, BaP, and pyrene according to wood materials (*n* = 3 for each wood material) are shown in Figure 1.

#### Wood operations

Total PAH concentrations in wood dust produced by three different power tools; saw, belt sander, and vibration sander using three wood types (fir, oak, and wood melamine) are shown in Table 2. Total PAH concentrations generated during vibration sanding were statistically higher than during belt sanding and sawing (*p*-value < 0.0001), while the latter two were similar. Moreover, separating the operations by wood material showed low total PAH concentrations (0.5–5.6 ppm) in dust for all operations from fir and oak. For wood melamine the total PAH concentrations were somewhat higher (1.7–7.9 ppm). All three wood working operations generated statistically significantly (*p* < 0.01) different total PAH, Σ6PAHs, BaP, and pyrene concentrations (Table 2).

**Table 2:** **Total PAHs, Σ6PAHs, BaP, and pyrene concentrations (ppm) measured in dust collected in the experimental chamber by wood type and operation**.

	***n***	**Total PAHs**		**Σ6PAHs**		**BaP**		**Pyrene**	
		**GM (GSD)**	**Diff**	**GM (GSD)**	**Diff**	**GM (GSD)**	**Diff**	**GM (GSD)**	**Diff**
**Operation^a^**		*p* < 0.0001		*p* = 0.001		*p* = 0.005		*p* = 0.002	
Sawing (A)	9	0.77 (1.83)	C	0.10 (2.33)	C	0.06^b^ (1.39)	C	0.26 (1.38)	C
Belt sanding (B)	9	0.69 (3.87)	C	0.12 (4.55)	C	0.11^b^ (2.56)		0.16 (3.52)	C
Vibration sanding (C)	9	3.39 (2.81)	A,B	0.84 (2.49)	A,B	0.10 (2.75)	A	0.78 (2.62)	A,B

n, number of samples; GM, geometric mean; GSD, geometric standard deviation; Diff, significantly different (Holm's multiplicity adjustment) GMs for the operations are indicated with the letters: A, sawing; B, belt sanding; C, vibration sanding.

a The following wood types were included fir, oak and wood melamine.

b Six of nine samples were non-detects.

### Field study

#### Personal inhalable dust monitoring

**Figure 2:**
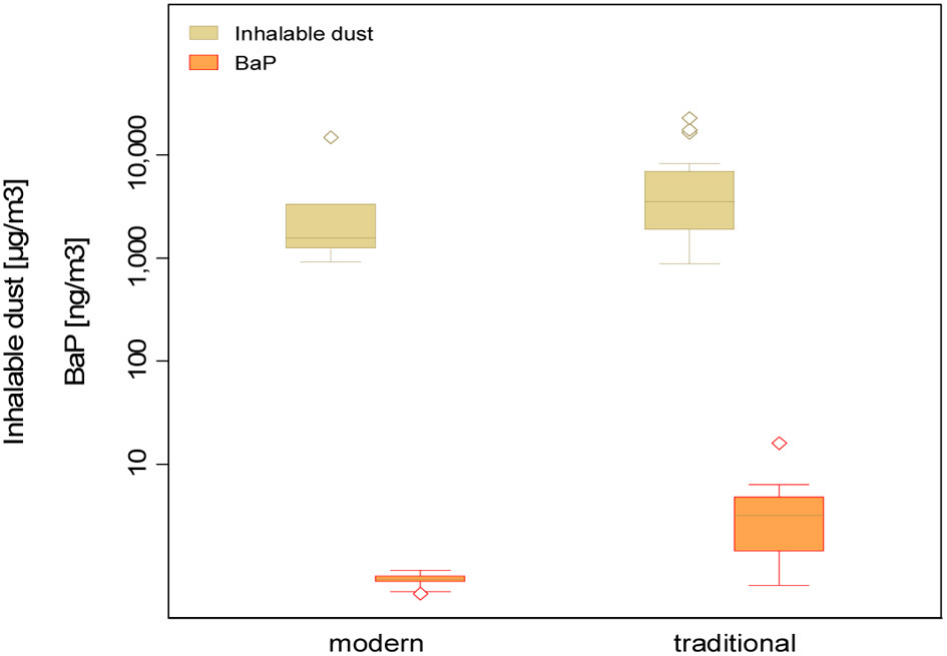
**Personal inhalable dust concentration (μg/m^3^) (*p*-value = 0.38) and BaP concentration (ng/m^3^) (*p*-value = 0.004) by factory type**.

PAH concentrations in personal inhalable dust samples collected among construction and furniture workers (*n* = 30) ranged from 37.5 to 119.8 ng m^−3^ (GM = 67.8 ng m^−3^ GSD = 1.4). A mixture of wood materials were used in both industries; fir, MDF, spruce, beech, and wood melamine. Table 3 summarizes by factory type and operation, the total PAHs, Σ6PAHs, BaP, and pyrene concentrations (ng m^−3^), as well as dust concentrations (μg m^−3^) quantified in the personal samples. Workers classified as “traditional factory workers” (*n* = 16) had statistically significantly higher (*p*-value = 0.004) BaP concentrations compared to the “modern furniture industry worker” (*n* = 14) (Figure 2); while the opposite was true for pyrene with concentrations almost four times higher for the modern factory (Table 3). No difference was detected for total PAHs (*p*-value = 0.54) and Σ6PAHs (*p*-value = 0.22).

**Table 3:** **Personal exposures to total PAHs, Σ6PAHs, BaP, and pyrene concentrations (ng m^−3^) found in inhalable wood dust, and inhalable dust concentrations (μg m^−3^) by factory type and operation**.

	***n***	**Total PAHs**	**Σ6PAHs**	**BaP**	**Pyrene**	**Inhalable dust**	
		**GM (GSD)**	**GM (GSD)**	**GM (GSD)**	**GM (GSD)**	**GM (GSD)**	**Diff**
**Factory Type**		*p* = 0.54	*p* = 0.22	*p* = 0.004	*p* = 0.004	*p* = 0.38	
Modern	14	65.43 (1.35)	8.94 (1.72)	0.75 (1.16)	9.45 (1.44)	2714 (3.95)	
Traditional	16	69.97 (1.42)	15.22 (1.70)	2.72 (2.51)	2.46 (3.19)	3860 (2.73)	
**Operation**		*p* = 0.07	*p* = 0.20	*p* = 0.06	*p* = 0.37	*p* = 0.02
Sawing (A)	13	74.07 (1.40)	15.96 (1.07)	2.89 (2.50)	2.67 (3.73)	6472 (3.73)	B,C
Sanding (B)	10	76.09 (1.29)	11.88 (1.72)	1.21 (2.24)	8.06 (1.34)	1771 (1.91)	A
Others (C)	7	55.79 (1.34)	8.08 (1.69)	0.73 (1.18)	6.35 (2.43)	2078 (2.34)	A

n, number of samples; GM, geometric mean; GSD, geometric standard deviation; Diff, significantly different (Holm's multiplicity adjustment) GMs for the operations are indicated with the letters: A, sawing; B, sanding; C, other.

We grouped wood operations into three categories; sanding, sawing, and others. “Others” included thin coating (varnish), planing, and assembly. No significant difference observed between wood operations for total PAHs (*p*-value = 0.07), Σ6PAHs (*p*-value = 0.20), BaP (*p*-value = 0.06) and for pyrene concentrations (*p*-value = 0.37) (Table 3).

Pyrene concentrations were low in all dust samples (GM = 11.3 ng m^−3^; GSD = 2.5), and detected in only 70% of the personal samples (*n* = 30). Although pyrene is not a carcinogen, its urinary metabolite 1-hydroxypyrene (1OHP) is used as a biomarker for exposures to PAHs. Thus, urinary 1OHP might not be a good indicator for PAH exposures from wood dust.

#### Personal dust concentration and particles size characterization

**Figure 3:**
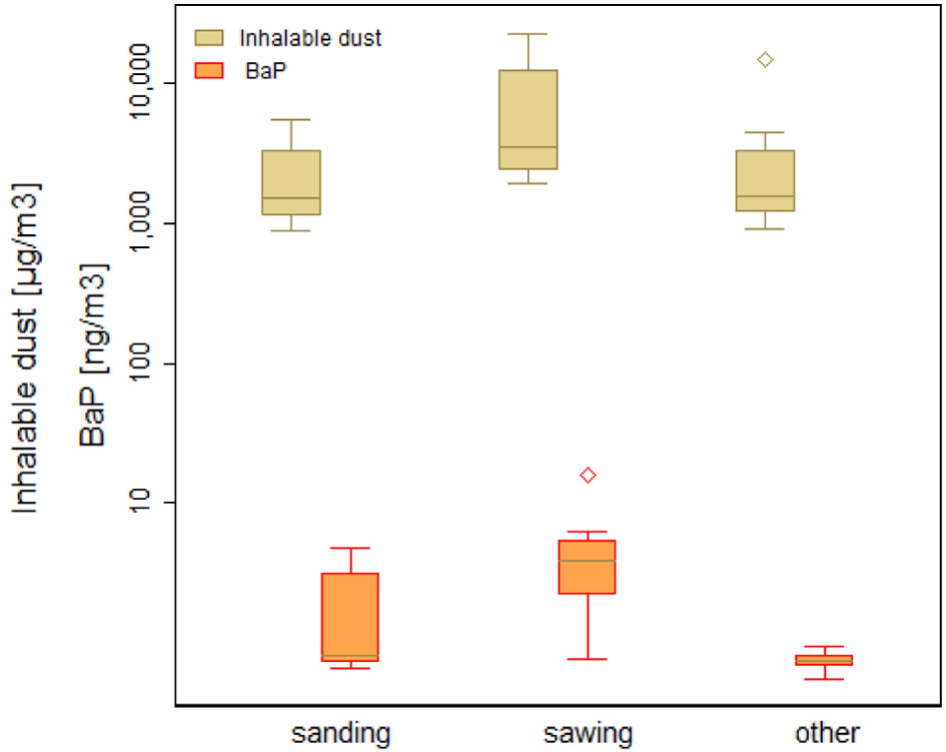
**Personal inhalable dust concentration (μg/m^3^) (*p*-value = 0.02) and BaP concentration (ng/m^3^) (*p*-value = 0.06) by operation**.

Personal inhalable dust concentrations varied greatly from 0.88 to 22.9 mg m^−3^ (*n* = 30) (GM = 2.8 mg m^−3^, GSD = 2.5). Overall, “traditional factory workers” (*n* = 16) were exposed to marginally but not statistically significantly (*p*-value = 0.38) higher total dust concentration compared to the “modern furniture industry workers” (*n* = 14) (Table 3). An outlier was observed for inhalable dust concentration with 168.13 mg m^−3^, which could be explained by projected coarse particles into the CFC sampler. Workplace exposures by operations (sanding, sawing, and other) showed the highest personal inhalable dust concentrations for sawing followed by others and sanding (Figure 3). There was a statistically significant difference in dust concentrations between different operations (*p*-value = 0.02) (Table 3).

**Figure 4:**
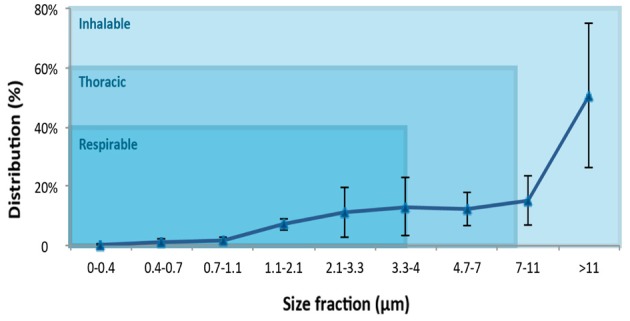
**Size distribution of wood dust particles in six wood processing factories (modern and traditional) with cut-off sizes according to a multi-stage cascade impactor**.

Total PAH concentrations for wood working operations were in the following descending order: sanding > sawing > others (Table 3). Wood dust particles size distributions by with the cutoff sizes indicated for each fraction for the six wood processing factories are presented graphically (Figure 4). Often the MMAD is used as an indicator of a particle's size in terms of its aerodynamic size. Thereby particles of differing geometric size, shape, and density are compared aerodynamically with the behavior of particles that are unit density (1 g/cm^3^) spheres. Dust deposits in various regions, and ACGIH has defined the fractions of the airborne particles as inhalable (100 μm cut-point), thoracic (10 μm cut-point), and respirable (4 μm cut-point) (ACGIH, 1994). Wood dust MMAD was measured to 10.15 μm (GSD = 1.53). Wood dust exposures were characterized by predominantly larges particles as already mentioned by Lee et al. (2011). Our results from wood processing factories (*n* = 6) support Lee et al.'s findings; low respirable dust concentration (25.4%) and high inhalable dust concentration, which corresponded to 65% of total sampled mass.

## Discussion

Wood workers are not only exposed to high concentrations of wood dust but also to low concentrations of PAHs (37.5–119.8 ng m^−3^) generated during wood processing operations. The distribution of PAHs and their concentrations varied depending on the type of wood industry (modern or traditional), wood materials, and woodworking operations.

PAH exposures during wood working operations in the plywood industry were first discussed by Kauppinen (1986). However, the authors could not determine the PAH source as the work environment included a forklift diesel engine, known to emit PAHs. In an earlier pilot study using two wood types (fir and oak) and vibration sanding, Huynh et al. (2009) quantified PAHs in generated wood dust. Compared to our study, the Huynh et al. (2009) study obtained twice the value for total PAH concentrations in dust generated from fir, and only half the value when sanding oak (Huynh et al., 2009). This could partially be explained by the different pieces of wood used (different trees), surface area sanded, duration, and the person conducting the experiment. However, taken together these results support the theory that PAHs are generated during wood operations with electric power tools.

Two parameters that greatly influenced PAH concentrations were the amount of resin in the wood material and the hardness of the wood. Using the same operation (vibration sanding), we measured the highest PAH concentrations with wood melamine (particle board), and the second highest with oak. For wood melamine, incomplete combustion of the plastic resin covering the particle board could explain the high PAH concentrations as none of the other wood materials had equally large amounts of resin available. Oak is very dense, and due to its hardness may produce more friction between the power tool and the wood fibers, creating increased heat, and consequently, more PAHs. An increase in temperature has shown to increase PAH concentrations and affected their mixture in studies of PAHs in asphalt (Cavallari et al., 2012). The generation of PAHs by incomplete combustion followed thermodynamic laws; where low temperatures gave light PAHs (2–3 aromatic ring structures) and with increasing temperatures all PAH types including the heavy PAHs (5–6 aromatic ring structures) increased. In our study, total PAH concentrations decreased in the following order: wood melamine > oak > fir > sipo > beech > MDF. It is interesting to note that MDF dust had low total PAHs and none carcinogenic. With respect to assessing wood workers' exposures, we therefore recommend to incorporate wood material types.

Regarding wood working operations, we observed that vibration sanding generated more PAHs compared to sawing and belt sanding in the experimental chamber. Again, we contributed this to more friction, thus more heat, created by the vibration sanding. Other studies corroborating the theory of PAHs generated during friction between the power tool and wood, can be gathered from other pyrolysis experiments such as carbonization of wood where PAH generation has already been described (Nakajima et al., 2007). They detected non-carcinogenic PAHs such as fluorene, phenanthrene, anthracene, and potentially carcinogenic PAH such as benz(a)anthracene, benzo(b)fluoranthene, benzo(k)fluoranthene, indeno(1,2,3-cd)pyrene, dibenz(a,h)anthracene and BaP in their samples. In our study, we obtained similar PAH profiles in the personal inhalable dust samples during wood working operations.

The wood working environment often includes simultaneous exposures to other substances (PAHs, formaldehyde, or wood preservatives). We have shown in our study the presence of PAHs in wood dust thus PAHs could potentially be “carried” by wood dust particles to where they deposit. Co-exposures to wood dust and PAHs therefore differ from co-exposures to wood dust and formaldehyde (a human carcinogen by IARC) in that PAHs are not just air borne (PAHs with less than five aromatic rings) as is the case for formaldehyde; but the dust acts as a vehicle for the heavier PAHs. Other similar particle-PAH exposures are found during occupational exposure to diesel exhaust where workers are co-exposed to diesel particles and PAHs. Recently, IARC classified diesel engine exhaust as carcinogenic to human (Group 1) (IARC, 2012). Lung cancer risk due to diesel engine exhaust exposure could partially be explained by the co-exposure to PAHs (Sauvain et al., 2003). These authors estimated that PAH exposure represented 3–13% of the lung cancer risk. In this diesel exhaust exposure study, BaP concentrations ranged between 0.25 and 4.53 ng m^−3^. Whereas similar BaP concentrations were obtained in our study among workers during wood working operations, the direct extrapolation to a cancer risk should be done with caution, as the matrix and the considered analytes were different in these two studies.

The exposure biomarker usually used to assess PAH exposures; urinary 1OHP, will probably be a poor indicator of PAH exposures from wood dust because pyrene concentrations were very low (0.51–25.3 ng m^−3^). Genotoxic effect biomarkers currently available are comet assays and micronucleus (MN) assays; however, none of these two are specific for wood dust. To establish effective strategies for prevention of avoidable occupational cancers, ideal biomarkers (exposure, response, and susceptibility biomarkers) should be developed specifically for wood dust toxicity in order to determine type of wood materials and operations that are mostly associated with increased cancer risk for early diagnosis and prevention of cancers.

In the six wood processing factories sampled, we found mostly large wood dust particles (>10 μm) as others have observed previously (Whitehead et al., 1981; Pisaniello et al., 1991; Harper et al., 2004; Lee et al., 2011). These large particles are known to be deposited in the sino-nasal region (NTP, 2000). Based on our results, we suggest that large wood particles with surface adsorbed PAHs can potentially play a role in the development of SNC. This hypothesis would better explain a co-exposure effect between large wood dust particles and PAHs than large wood dust particles and formaldehyde because wood dust was found to be a poor vehicle for formaldehyde, which existed mainly in vapor form (Gosselin et al., 2003). A recent study corroborates our hypothesis as it did not show any association between formaldehyde exposures and increasing risk of SNCs among wood workers (Krief et al., 2008).

Wood dust exposure concentrations were determined by 37 mm CFC sampler in our study. This sampler is widely used by American industrial hygienists (Harper and Muller, 2002), and its use is also recommended in France (Association Française De Normalisation, 2008). However, the CFC sampler has shown to significantly underestimate the total inhalable fraction when large particles were present (Kenny et al., 1997). Wood dust particles are often large particles as observed in our study. We observed relatively high exposures to wood dust among our construction and furniture workers (GM 2.8 mg m^−3^, GSD 2.5). Indeed, 93% of workers surveyed in our study had total dust concentrations above the TLV recommended by ACGIH and the French OEL (1 mg m^−3^); and 53% were above the Swiss OEL (2 mg m^−3^).

Nasal cancer rates have been fairly constant showing an increased risk associated with exposure to wood dust (Pukkala et al., 2009). In a fairly recent and exceptionally large (*n* = 15 million people) Nordic occupational epidemiologic study, an observed standardized incidence ratio (SIR) of 1.84 (95%CI 1.66–2.04) was observed for men and 1.88 (95%CI 0.90–3.46) for women woodworkers (Pukkala et al., 2009). For nasal adenocarcinoma, the SIR in men was very high 5.50 (4.60–6.56). In an earlier study (Kauppinen et al., 2006), the mean wood dust concentration among Nordic construction wood workers was estimated to 1.5–3.3 mg m^−3^ (GM). This estimate is close to the mean wood dust concentrations (GM 2.8 mg m^−3^, GSD 2.5) observed in our study, thus exposures have not declined significantly the last decade, and we can therefore not expect the SNC rates to decline. Other authors (Lee et al., 2011) suggest that wood dust exposure levels may have decreased over recent decades possibly due to the changes in equipment, production methods, and upgrading engineering ventilation systems for dust control (Teschke et al., 1999; Galea et al., 2009). Albeit the overall wood dust concentrations were similar to older studies, we do find some support for decreased wood dust concentrations in modern factories with improved occupational hygiene. In our study, traditional factories had higher total wood dust concentrations compared to modern factories, but the difference was not statistically significant, probably due to low statistical power. Future epidemiology studies should collect information regarding modern and traditional factories as this could impact the cumulative exposure estimates.

## Conclusion

Wood workers are not only exposed to high concentrations of wood dust but also to low concentrations of PAHs (37.5–119.8 ng m^−3^). The PAH concentrations and mixtures varied depending on the type of wood industry (modern or traditional), wood materials followed by woodworking operations. Future epidemiology studies would benefit from collecting this information as it could impact the cumulative exposure estimates. Effective strategies for prevention of SNC among wood workers would be to develop specific wood dust toxicity biomarkers (exposure, response, and susceptibility biomarkers) in order to determine type of wood materials and operations associated with increased cancer risk; and for surveillance of wood workers for early diagnosis and prevention of cancers. Future toxicological studies should explore a possible SNC mechanism involving nasal mucosa irritation from chronic exposures to large wood dust particles deposited in the nasal cavity and co-exposure to PAHs.

### Conflict of interest statement

The authors declare that the research was conducted in the absence of any commercial or financial relationships that could be construed as a potential conflict of interest.
